# Supplementary material for: Mathematical modeling and performance evaluation of Ducted Horizontal-axis Helical Wind Turbines: Insights into aerodynamics and efficiency
Source: PLoS One. 2024 Jun 17;19(6):e0303526. doi: 10.1371/journal.pone.0303526 (PMC11182528; doi:10.1371/journal.pone.0303526)
Supplement: Attachment — Submitted filename: PONE-D-23-38272.pdf [file pone.0303526.s002.pdf]

PLOS ONE

Mathematical Modeling and Performance Evaluation of Ducted Horizontal-axis Helical Wind Turbines: Insights into Aerodynamics and Efficiency

--Manuscript Draft--

**Manuscript Number:**	PONE-D-23-38272
**Article Type:**	Research Article
**Full Title:**	Mathematical Modeling and Performance Evaluation of Ducted Horizontal-axis Helical Wind Turbines: Insights into Aerodynamics and Efficiency
**Short Title:**	Aerodynamics and Efficiency of Ducted Helical Wind Turbines: A Mathematical Analysis
**Corresponding Author:**	Zishan Shaikh<br>Universiti Kebangsaan Malaysia Institut Penyelidikan Tenaga Suria<br>Bangi, Selangor MALAYSIA
**Keywords:**	Wind Energy; CFD; Helical Wind Turbine; Blade Element Actuator Disk Method; ANN
**Abstract:**	Decentralized, small energy infrastructure is getting more attention as energy demand rises. New turbines make them successful. However, many of these turbines do not follow many of the basic ideas established to evaluate their performance, leaving no precise technique or mathematical model. This research developed a Ducted Horizontal-axis Helical Wind Turbine (DHAHWT). The DHAHWT is a duct-mounted helical savonius turbine with a venturi and diffuser to improve flow. Unlike a vertical axis helical savonius turbine, DHAHWT revolves roughly parallel to the wind, making it a horizontal turbine. This complicates mathematical and theoretical analysis. This study created a DHAHWT mathematical model. COMSOL simulations utilizing Menter's Shear Stress Transport model (SST) at an incoming velocity range of 1m/s to 4m/s were used to evaluate the turbine's interaction with the wind. MATLAB was used to train an artificial neural network (ANN) utilizing COMSOL data to obtain greater velocity data. The Mean Average Percentage Error (MAPE) and Root Mean Square Error (RMSE) of ANN data was 3%, making it very accurate. Further, using advanced statistical methods the Pearson's correlation coefficient was calculated resulting in a better understanding of the relationship of between the incoming velocity and the velocity at different sections of the wind turbine. This study will shed light on the aerodynamics and working of DHAHWT.
**Order of Authors:**	Zishan Shaikh
	Ahmad Fazlizan
	Halim Razali
	Kok Hoe Wong
	Altaf Hossain Molla
	Rabiu Aliyu Abdulkadir
	Dumitru Baleanu
	Rabha W. Ibrahim
**Additional Information:**	
**Question**	**Response**
**Financial Disclosure**<br><br>Enter a financial disclosure statement that describes the sources of funding for the work included in this submission. Review the submission guidelines for detailed	The author(s) received no specific funding for this work.

Powered by Editorial Manager® and ProduXion Manager® from Aries Systems Corporation

requirements. View published research articles from *PLOS ONE* for specific examples.<br><br>This statement is required for submission and **will appear in the published article** if the submission is accepted. Please make sure it is accurate.<br><br>**Funded studies**<br>Enter a statement with the following details:<br>• Initials of the authors who received each award<br>• Grant numbers awarded to each author<br>• The full name of each funder<br>• URL of each funder website<br>• Did the sponsors or funders play any role in the study design, data collection and analysis, decision to publish, or preparation of the manuscript?<br><br>Did you receive funding for this work?	
**Competing Interests**<br><br>Use the instructions below to enter a competing interest statement for this submission. On behalf of all authors, disclose any competing interests that could be perceived to bias this work—acknowledging all financial support and any other relevant financial or non-financial competing interests.<br><br>This statement is required for submission and **will appear in the published article** if the submission is accepted. Please make sure it is accurate and that any funding sources listed in your Funding Information later in the submission form are also declared in your Financial Disclosure statement.<br><br>View published research articles from *PLOS ONE* for specific examples.	The authors have declared that no competing interests exists.

Powered by Editorial Manager® and ProduXion Manager® from Aries Systems Corporation

**NO authors have competing interests**<br><br>Enter: *The authors have declared that no competing interests exist.*<br><br>**Authors with competing interests**<br><br>Enter competing interest details beginning with this statement:<br><br>*I have read the journal's policy and the authors of this manuscript have the following competing interests: [insert competing interests here]*<br><br>* typeset	
**Ethics Statement**<br><br>Enter an ethics statement for this submission. This statement is required if the study involved:<br><br>• Human participants<br>• Human specimens or tissue<br>• Vertebrate animals or cephalopods<br>• Vertebrate embryos or tissues<br>• Field research<br><br>Write "N/A" if the submission does not require an ethics statement.<br><br>General guidance is provided below. Consult the submission guidelines for detailed instructions. **Make sure that all information entered here is included in the Methods section of the manuscript.**	N/A

Powered by Editorial Manager® and ProduXion Manager® from Aries Systems Corporation

**Format for specific study types**<br><br>**Human Subject Research (involving human participants and/or tissue)**<br>• Give the name of the institutional review board or ethics committee that approved the study<br>• Include the approval number and/or a statement indicating approval of this research<br>• Indicate the form of consent obtained (written/oral) or the reason that consent was not obtained (e.g. the data were analyzed anonymously)<br><br>**Animal Research (involving vertebrate animals, embryos or tissues)**<br>• Provide the name of the Institutional Animal Care and Use Committee (IACUC) or other relevant ethics board that reviewed the study protocol, and indicate whether they approved this research or granted a formal waiver of ethical approval<br>• Include an approval number if one was obtained<br>• If the study involved *non-human primates*, add *additional details* about animal welfare and steps taken to ameliorate suffering<br>• If anesthesia, euthanasia, or any kind of animal sacrifice is part of the study, include briefly which substances and/or methods were applied<br><br>**Field Research**<br><br>Include the following details if this study involves the collection of plant, animal, or other materials from a natural setting:<br>• Field permit number<br>• Name of the institution or relevant body that granted permission	
**Data Availability**<br><br>Authors are required to make all data underlying the findings described fully available, without restriction, and from the time of publication. PLOS allows rare exceptions to address legal and ethical concerns. See the PLOS Data Policy and FAQ for detailed information.	Yes - all data are fully available without restriction

A Data Availability Statement describing where the data can be found is required at submission. Your answers to this question constitute the Data Availability Statement and **will be published in the article**, if accepted.<br><br>**Important:** Stating 'data available on request from the author' is not sufficient. If your data are only available upon request, select 'No' for the first question and explain your exceptional situation in the text box.<br><br>Do the authors confirm that all data underlying the findings described in their manuscript are fully available without restriction?	
**Describe where the data may be found in full sentences. If you are copying our sample text, replace any instances of XXX with the appropriate details.**<br><br>• If the data are **held or will be held in a public repository**, include URLs, accession numbers or DOIs. If this information will only be available after acceptance, indicate this by ticking the box below. For example: *All XXX files are available from the XXX database (accession number(s) XXX, XXX.).*<br>• If the data are all contained **within the manuscript and/or Supporting Information files**, enter the following: *All relevant data are within the manuscript and its Supporting Information files.*<br>• If neither of these applies but you are able to provide **details of access elsewhere**, with or without limitations, please do so. For example:<br><br>*Data cannot be shared publicly because of [XXX]. Data are available from the XXX Institutional Data Access / Ethics Committee (contact via XXX) for researchers who meet the criteria for access to confidential data.*<br><br>*The data underlying the results presented in the study are available from (include the name of the third party*	All relevant data are within the manuscript and its Supporting Information files.

and contact information or URL).<br>• This text is appropriate if the data are owned by a third party and authors do not have permission to share the data.<br><br>* typeset	
Additional data availability information:	Tick here if your circumstances are not covered by the questions above and you need the journal’s help to make your data available.

Click here to access/download;Manuscript;PlosOne Paper.docx

Mathematical Modeling and Performance Evaluation of Ducted Horizontal-axis Helical Wind Turbines: Insights into Aerodynamics and Efficiency

Shaikh Zishan[1], Ahmad Fazlizan[1,*], Halim Razali[1], Kok Hoe Wong[2], Altaf Hossain Molla[3], Rabiu Aliyu Abdulkadir[4,5], Dumitru Baleanu[6,7,8*], Rabha W. Ibrahim[6,9,10]

[1] Solar Energy Research Institute, Universiti Kebangsaan Malaysia, 43600 Bangi, Selangor, Malaysia.

[2] Carbon Neutrality Research Group (CNRG), University of Southampton Malaysia, Iskandar Puteri 79200, Malaysia.

[3] Department of Mechanical and Manufacturing Engineering, Faculty of Engineering and Built Environment, Universiti Kebangsaan Malaysia, 43600 Bangi, Selangor, Malaysia.

[4] Faculty of Information Science and Technology, Universiti Kebangsaan Malaysia, 43600 Bangi, Selangor, Malaysia.

[5] Department of Electrical Engineering, Aliko Dangote University of Science and Technology, Wudil, Nigeria.

[6]Department of Computer Science and Mathematics, Lebanese American University, Beirut, Lebanon

[7]Institute of Space Sciences, R76900 Magurele-Bucharest, Romania

[8]Department of Medical Research, China Medical University,Taichung 40402, Taiwan

[9]Information and Communication Technology Research Group, Scientific Research Center, Al-Ayen University, Thi-Qar, Iraq

[10]Near East University, Mathematics Research Center, Department of Mathematics, Near East Boulevard, PC: 99138, Nicosia /Mersin 10-Turkey

Emails: a.fazlizan@ukm.edu.my dumitru.baleanu@gmail.com, rabhaibrahim@yahoo.com

Abstract: Decentralized, small energy infrastructure is getting more attention as energy demand rises. New turbines make them successful. However, many of these turbines do not follow many of the basic ideas established to evaluate their performance, leaving no precise technique or mathematical model. This research developed a Ducted Horizontal-axis Helical Wind Turbine (DHAHWT). The DHAHWT is a duct-mounted helical savonius turbine with a venturi and diffuser to improve flow. Unlike a vertical axis helical savonius turbine, DHAHWT revolves roughly parallel to the wind, making it a horizontal turbine. This complicates mathematical and theoretical analysis. This study created a DHAHWT mathematical model. COMSOL simulations utilizing Menter's Shear Stress Transport model (SST) at an incoming velocity range of 1m/s to 4m/s were used to evaluate the turbine's interaction with the wind. MATLAB was used to train an artificial neural network (ANN) utilizing COMSOL data to obtain greater velocity data. The Mean Average Percentage Error (MAPE) and Root Mean Square Error (RMSE) of ANN data was 3%, making it very accurate. Further, using advanced statistical methods the Pearson's correlation coefficient was calculated resulting in a better understanding of the relationship of between the incoming velocity and the velocity at different sections of the wind turbine. This study will shed light on the aerodynamics and working of DHAHWT.

Keywords: Wind Energy, CFD, Helical Wind Turbine, Blade Element Actuator Disk Method, ANN

1. INTRODUCTION

One of the great difficulties of the twenty-first century is ensuring reliable, economical, and environmentally sustainable energy supplies. Energy infrastructure is at the heart of this dilemma, serving as a point of convergence for a wide range of policy objectives ranging from economic growth and national security to climate change mitigation and social inequity[1]. As the demand in energy increases in the world a surge in the demand for small and more efficient energy systems is seen. Similar is the case in the field of wind energy[2]. Many researchers have worked on a similar aim and introduced systems that can help in achieving the same. Some have worked on adding a duct in order to increase the mass flow of air through the turbine, like initial development done by Sabzevaria in 1977 [3] and further development to the latest wind lens by Yuji Ohya and Takashi Karasudani in 2010 [4], Chong et al. Omnidirectional Guided Vanes (ODGV) [5] and to the latest INVELOX, studied by by Allaei et al. in 2014 [6] and further developed by Wanlong Han et al. in 2015 [7]. Further some researchers have taken another interesting way, including wind energy recovery from manmade sources of wind [5,8–14], introducing novel and biomimetic blades [15,16], Smart blades [17], or bladeless turbines designed by corporations like Magenn Power (Lighter than air) and Vortex Bladeless Turbine (Skybrator). All these efforts have aided in the increase in efficiency with a significant reduction in size and noise.

Apart from the above-mentioned benefits these systems have also changed the way different empirical and semi-empirical models would fit the analysis of the system. In 2007 Van Bussel [18] had developed a theoretical model based on Betz's theory suggesting a direct relationship between power augmentation and mass flow increase. Hansen [19] proved that the power coefficient increase is proportional to the increase in the ratio of the mass flow through the shrouded turbine to that through the bare turbine. Apart from the above-mentioned theories there are many theories that are used in the evaluation of the performance of a wind turbine. These models are categorized into two types: semi-empirical models for the near wake and semi-empirical models for the far wake. Their categorization is based on their effectiveness in assessing the aerodynamic efficiency of blades and forecasting the flow pattern in the distant wake zone. Semi-empirical models take into account the forces exerted on the rotor, aiding in power output prediction. Additionally, they consider blade shape, which directly influences both aerodynamic blade performance and the wake near the rotor region.

A simple model in the under semi-empirical near wake models is the Blade element momentum which suggests the calculation of the load on the turbine by dividing the turbines into small sections [20]. An extension of the same is the actuator disc theory, which suggests that a blade is a semi-permeable disk at the rotor plane and the downwash velocity from the turbine reduces roughly by 66% when compared to the free stream velocity. The actuator disk theory when coupled with

Computational Fluid Dynamics (CFD) can help calculate the flow-field in the wake [21]. However, these theories are unable calculate the output for certain novel turbines with a long central axis and a velocity gradient within the turbine and multiple types of wind velocities.

This paper aims at deriving a novel method for the calculation of power output which would consider the different forms of velocities within the turbine range as well as the velocity gradient throughout the turbine. Figure 2 talks about the outline and the overview of the testing methods employed in order to reach the conclusion. This is necessary as it would help in understanding the not just the overall performance but also the wake behavior related change in efficiency. The proposition made in this research paper is based on the data collected from CFD simulation in stationary state, which was further used in Artificial Neural Network (ANN) to generate more data and finally evaluated for Pearsons's coefficient.

2. THEORY REVIEW

The status quo for the mechanical power generation from a wind turbine is based on the Betz theory (1926). This model was developed based on linear momentum theory. Linear Momentum theory is based on the analysis of loading on the blades by application of conservation of linear and angular momentum theories. For any analysis of a turbine performance, it is necessary that a control volume is assumed, in which the boundaries resemble the shape of a stream tube. The status quo is suggested to be a general model that applicable for various wind turbines. The assumptions used were as follows:

- Control volume of the fluid
- Homogenous, incompressible steady state fluid flow
- No frictional drag
- An infinite number of blades
- Uniform thrust over the disk or rotor areas.
- Continuity of velocity through the disk
- A non-rotating wake
- The static pressure far upstream and far downstream is equal to the undisturbed ambient static pressure.

Utilizing the principle of preserving linear momentum within the control volume encompassing the entire system allows for the determination of the overall force acting on the contents within the control volume. In adherence to the law of mass conservation, the quantity of air passing through various segments of the stream tube remains consistent. [22]. Since kinematic models, often referred to as explicit models, are straightforward wake models that can be solved analytically, they are appropriate for assessing the wind speed deficiencies in sizable wind farms. These models simulate the velocity deficit of the wake behind a turbine using the momentum equation. The wake's initial expanding zone is not considered in the wake descriptions. The kinematic models of Jensen, Larsen (first order and second order), and Frandsen are four of the most well-known [23,24] are talked about in table 1. The development of wake is shown in figure 1. These models rely on the initial velocity profile of the near-wake region to calculate the velocity deficit and wake expansion using the simplified momentum equations based on these velocity profiles. To consider, the change in the intensity of turbulence these models must be coupled with a turbulence model as the

original forms of the models cannot evaluate such advance modelling. The Larsen model, one of the previously mentioned models, can consider the impact of ambient turbulence on wake expansion. The governing equations needed for each of these kinematic far-wake models are the basis of the simulations. In comparison to a boundary layer that is highly turbulent, a laminar ABL has longer far-wake areas and a higher velocity deficit. Lower energy is available to the wind turbines further downstream because of the longer wake. However, due to the fluctuating stresses that come from increased turbulence, this may also increase the rate of turbine blade wear. On the other side, increased turbulence intensity speeds up wake recovery [25].

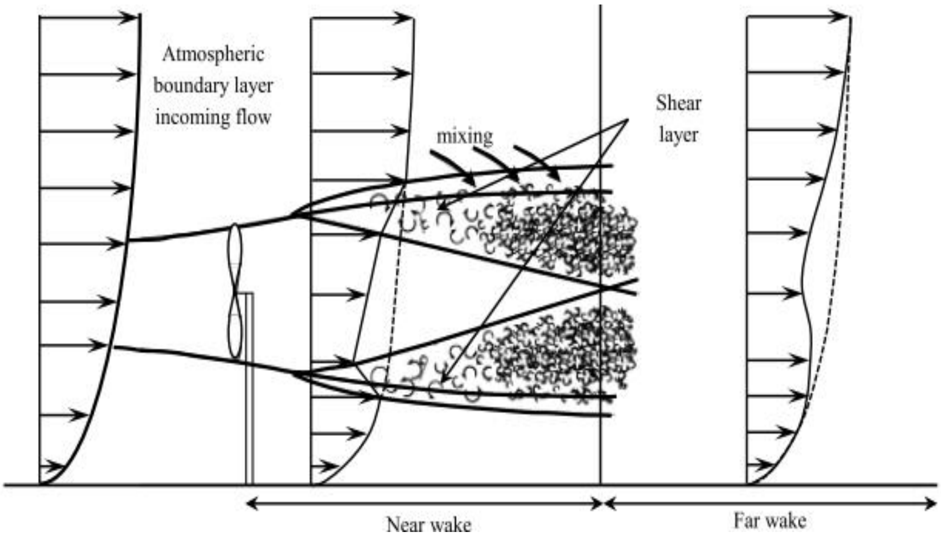

FIGURE 1: Schematic representation of wind turbine wake when turbine is placed in an Atmospheric Boundary Layer [26].

The development of Diffuser-Augmented Wind Turbines (DAWTs) has spanned over 50 years without commercial success. Initially, the conversion process was poorly understood, and experimentalists focused on maximizing pressure drop over the rotor disk without theoretical guidance. Increasing the diffuser area and negative back pressure at the exit proved beneficial. Claims of performance enhancements by a factor of 4 or more were unconfirmed. A simple momentum theory showed that power augmentation in DAWTs is proportional to mass flow increase at the nozzle, achievable by adjusting the diffuser exit ratio and reducing negative back pressure. The theory predicts an optimal pressure drop of 8/9, the same as for bare wind turbines, with maximum energy extraction per unit of volume. Practical experiments have not achieved power augmentation factors above 3, although theoretical rotor power coefficients of CP, rotor = 2.5 may be achievable at the cost of larger diffuser area ratios (typically $\beta > 4.5$).

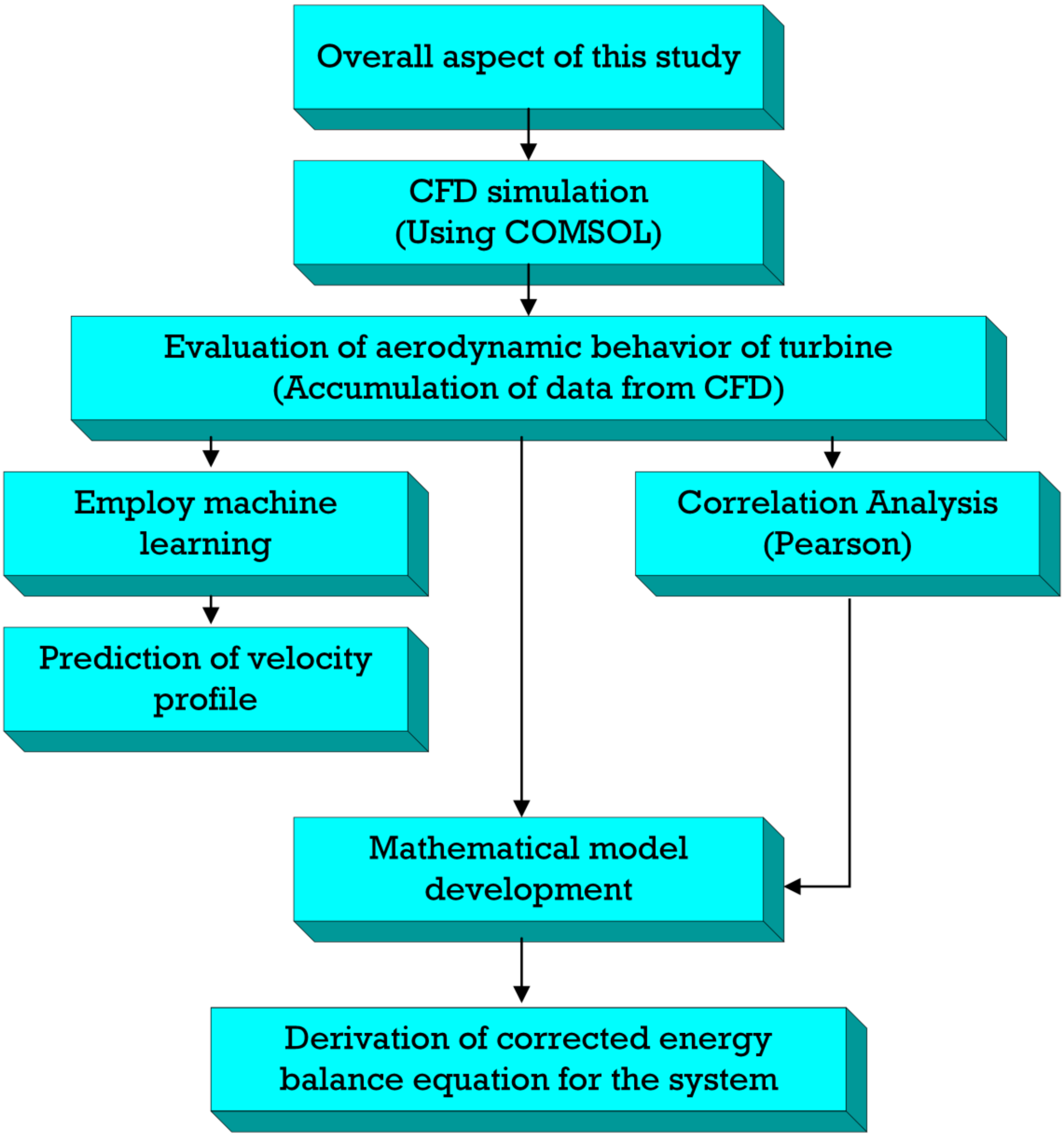

FIGURE 2: Flow of study and methodology

3. METHODOLOGY

3.1. Energy Balance For DAWT

The change in energy for an open system of flow of fluid through a duct with isothermal conditions and no work done is given by:

$$\Delta E = \frac{P_1}{\rho} + \frac{V_1^2}{2} - \frac{P_2}{\rho} + \frac{V_3^2}{2} \quad \text{(i)}$$

To calculate the change in energy when work is done can be calculated by combining equation 1 and wind energy equation.

$$\Delta E = \frac{P_1}{\rho} + \frac{V_1^2}{2} - \left[\left(\frac{P_2}{\rho} + \frac{V_3^2}{2}\right) + \frac{1}{2}\rho A V_1^3\right] \text{.........} \quad \text{(ii)}$$

Considering the hypothesis of Actuator disk theory, we get.

$$\Delta E = \frac{P_1}{\rho} + \frac{V_1^2}{2} - \left[\left(\frac{P_2}{\rho} + \frac{(V_1 - 0.66(V_1))^2}{2}\right) + \frac{1}{2}\rho A V_1^3\right] \text{........(iii)}$$

Considering the hypothesis of One-dimensional momentum theory, we get.

$$\Delta E = \frac{P_1}{\rho} + \frac{V_1^2}{2} - \left[\left(\frac{P_2}{\rho} + \frac{(\gamma(1-a)V_0)^2}{2}\right) + \frac{1}{2}\rho A V_1^3\right] \text{..............(iv)}$$

As per the actuator disk theory, the velocity of the wind flow drops by $\frac{2}{3}$ (66.66%) after the wind turbine. Further, as per the one-dimensional momentum theory in a DAWT this drop may be less as the optimum axial induction factor is $\frac{1}{3}$. However, no matter how small the drop in the velocity it would significantly reduce the exit velocity and cause a negative impact on the Energy Recovery System causing major failure and other issues.

3.2. DHAHWT Wind Turbine

The DHAHWT denotes a ducted helical wind turbine characterized by wind flow alignment along its central axis. This configuration classifies the turbine as a horizontal axis wind turbine due to wind direction. Despite previous employment as a vertical axis wind turbine in open natural wind conditions, this marks its inaugural utilization within a duct in a horizontal axis orientation.

The innovative implementation of this system aims to exploit hitherto neglected wind energy resources generated by anthropogenic sources. Conventional wind turbine designs are unsuitable for this purpose, as their application could compromise system efficiency. Thus, the necessity for an inventive turbine design with distinct aerodynamic properties becomes evident.

In instances involving a novel wind turbine design that deviates from prevalent assumptions, the applicability of currently employed equations may exhibit limitations in accurately capturing the turbine's operational outcomes. Subsequent sections will delve into the intricate wind behaviour in the vicinity of the turbine. A comprehensive comprehension of this phenomenon mandated the execution of a steady-state CFD investigation for the DHAHWT.

To facilitate the simulation, the Reynolds Averaged Navier-Stokes Equation (RANS) was employed, utilizing Menter's Shear Stress Model (SST). The choice of RANS stemmed from the study's objective to grasp the cumulative effects arising from wind-turbine interaction, transcending mere eddy current observations. Similarly, the selection of SST was predicated on its amalgamation of k-ε and k-ω turbulence models, yielding enhanced visualization of both wall-bounded and free-stream flow characteristics.

For a given simulation it is necessary to have an appropriate y^+ value which is a dimensionless parameter. This dimensionless value helps to classify the types of boundary layers. y^+ is given by

$$y^+ = \frac{u.y}{v}$$

Where, u is the shear velocity at the nearest wall, y is the absolute cell distance from the nearest wall and v is the kinematic viscosity. A value of y^+ less than 5 is considered as sub laminar. However, a y^+value of more than 5 is excellent for 3D visualization of simulation.

3.3. Simulation Analysis

A Boundary condition is the interaction between the system and the environment. The boundary conditions in a fluid dynamics study include velocity inlet and outlet, wall (no-slip/slip) etc. For the topic of study following were the boundary conditions.

- The domain of the turbine was selected to be solid.
- The domain of the duct was selected to be non-solid.
- The fluid material selected was Air of equilibrium discharge.
- Properties of the fluid were user defined to 30○ Celsius.
- The material at the walls of the duct was selected as Polyvinyl Chloride (PVC).
- At the wall, NO SLIP condition was applied.
- Inlet and outlet specified.
- Outlet pressure was set to adjust in accordance with the ambient pressure to account for the back pressure.
- Inlet velocity was for range 1 m/s to 4 m/s.

The simulation was carried out considering the flow to be incompressible range hence the continuity equation would apply [27].

$$\rho(\mathbf{u}\cdot\nabla)\mathbf{u} = \nabla\cdot[-\rho\mathbf{I} + \mathbf{K}] + \mathbf{F} \qquad \text{(v)}$$

$$\rho\nabla.\boldsymbol{u} = 0 \qquad \text{(vi)}$$

Here, 'u' is the fluid velocity, p is the fluid pressure and ϱ is the fluid density. 'F' is the external forces that are fluid. However, in equation 1 no additional forces have been added hence F can be neglected. The value of K in equation 1 is given by:

$$\mathbf{K} = (\mu + \mu_{\mathrm{T}})(\nabla\mathbf{u} + (\nabla\mathbf{u})^{\mathrm{T}}) \qquad \text{(vii)}$$

$$\rho(\mathrm{u}\cdot\nabla)\mathrm{k} = \nabla\cdot[(\mu + \mu_{\mathrm{T}}\sigma_k)\nabla\mathrm{k}] + P - \beta_0^*\rho\omega k \qquad \text{(viii)}$$

$$\rho(\boldsymbol{u}\cdot\nabla)\omega = \nabla\cdot[(\mu + \mu_T\sigma_\omega)\nabla\omega] + \frac{\gamma}{\mu_T}\rho P - \rho\beta_0\omega^2 + 2(1 - f_{v1})\frac{\sigma_{\omega 2}\rho}{\omega}\nabla k\cdot\nabla\omega, \quad \omega = om \qquad \text{(ix)}$$

Here, 'om' is the specific dissipation rate and 'k' is the turbulent kinetic energy.

$$\nabla G\cdot\nabla G + \sigma_w G(\nabla\cdot\nabla G) = (1 + 2\sigma_w)G^4, \quad \ell_\omega = \frac{1}{G}\cdot\frac{\ell_{ref}}{2} \qquad \text{(x)}$$

Here, 'G' is the reciprocal wall distance.

$$\mu_T = \rho\frac{a_1 k}{max(a_1\omega, Sf_{v2})}, S = \sqrt{2\boldsymbol{S}:\boldsymbol{S}}, \ \boldsymbol{S} = \frac{1}{2}(\nabla\boldsymbol{u} + (\nabla\boldsymbol{u})^T) \qquad \text{(xi)}$$

Figure 3 shows the diagram of the setup drawn in COMSOL and meshed. For the meshing of the setup a physics-controlled mesh was generated. This was the case as it can generate the best meshing with the negligible error in the meshing. Further a mesh independent study was carried out to rule of the influence of the size of the mesh on the results generated. Here, the minimum element size is 0.9, maximum element size is 13.8 Maximum element growth rate being 1.08. The overall mesh quality was 0.234. A mesh independent study was conducted to rule out the possibility of results being dependent on the mesh size. Further a grid convergence study suggests that the error is lower than 10^{-1}, which is an industrially acceptable result. This can be seen in Figure 4.

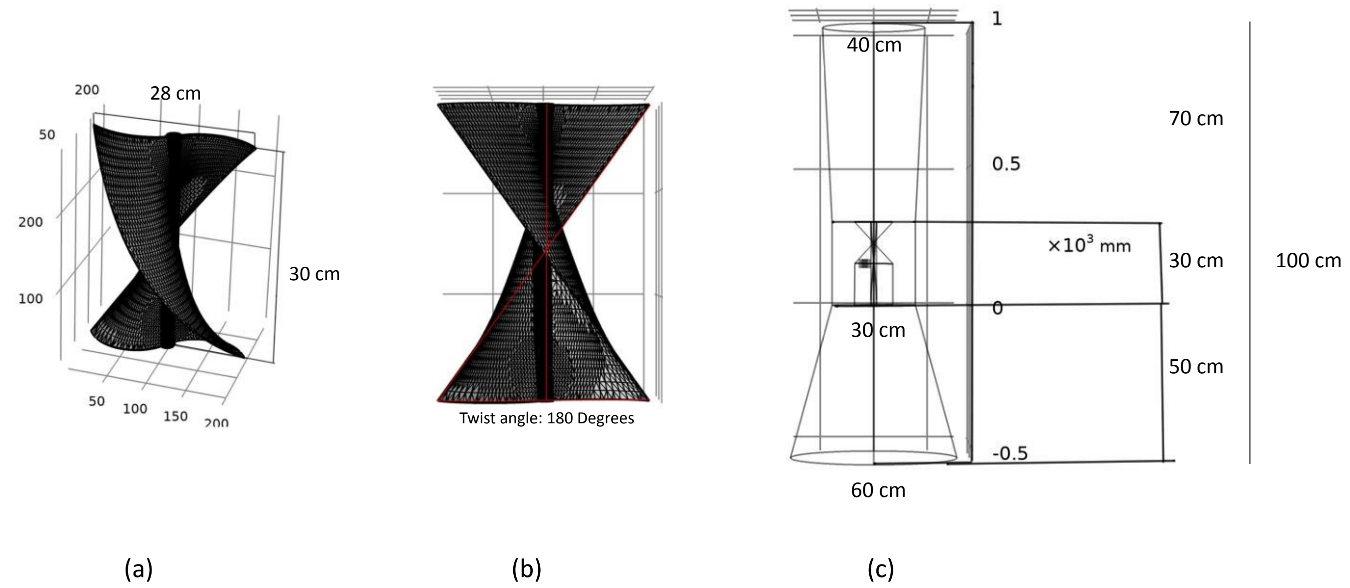

FIGURE 3: (a) The meshing and geometry of the helical turbine (b) Meshing and twist angle of the turbine in COMSOL (c) Geometry of the complete system.

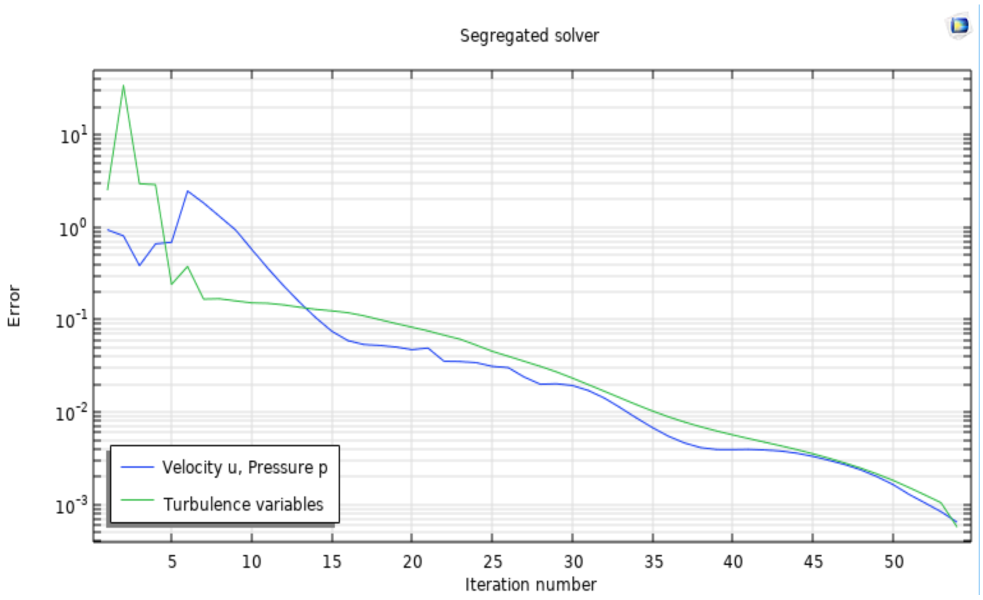

FIGURE 4: Error Study

3.4. Data Collection and Processing

ANN being a versatile tool, has also found its use in the field of wind energy research. Acarer et al. evaluated the use of radial wind turbines (RWT) in 2020 [28]. The idea was that the design would be easily adaptable to residential uses. An exterior casing serves as the entry point for the airflow, which interacts tangentially with the turbine blade before leaving the casing through a chimey. In 2018, Wang et al. used a suggested design with preliminary optimizations utilising CFD simulations and response surface modelling with applications of machine learning algorithms [29]. Additionally, Sarkic-Glumac et al. conducted meta-modelling with assistance from an artificial neural network (ANN) to optimise the shape. Astonishingly, the 3D model's Cp value of 0.29 at = 0.55 indicated a 103% increase in Cp over the designs that had previously recorded the maximum efficiency [30]. In this work the evaluation of wind profile around the is evaluated at various velocities. For the training set velocity data range from 1m/s to 3.5 m/s was evaluated. This data was obtained from COMSOL simulation. Further, the data of 4m/s velocity was used as a test range and later the data for 6m/s velocity was predicted.

All the basic training data set was imported from the simulations conducted in COMSOL. The data collected was at different points on the turbine (6 points across the diameter and 30 along the axis) at velocities ranging from 1m/s to 3.5m/s. The points can be seen in figure 5 Further, to evaluate the testing, the data obtained from COMSOL was compared with the prediction data obtained from ANN. The data was further evaluated for MAPE, RMSE and MBE. The evaluation for the same was based on the following equations. The evaluation of these statistical methods is done using the following equations:

$$MAPE = \frac{1}{n}\sum_{i=1}^{n}\frac{V_{sim} - V_{ANN}}{V_{sim}}$$

$$MAPE = \sqrt{MAPE = \frac{1}{n}\sum_{i=1}^{n}(V_{sim} - V_{ANN})^2}$$

$$MBE = \frac{1}{n}\sum_{i=1}^{n}(V_{sim} - V_{ANN})^2$$

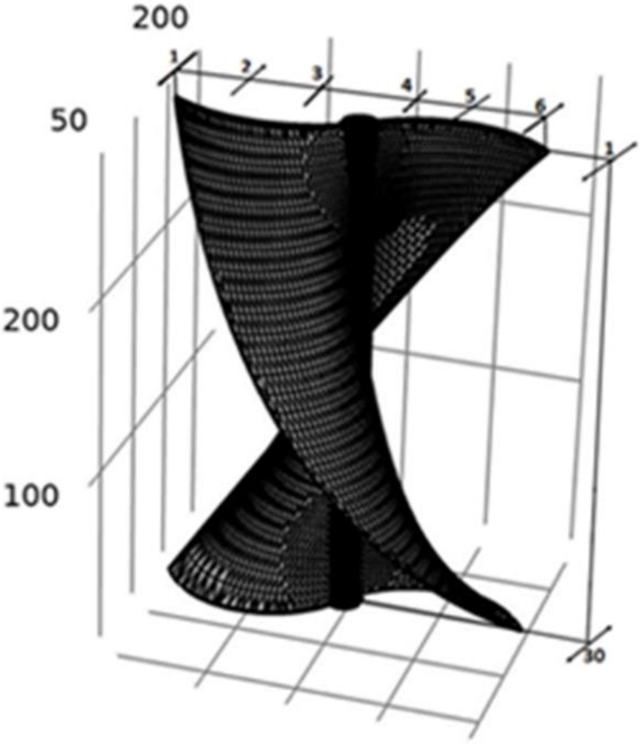

FIGURE 5: 6 Points across the diameter and 30 points along the axis for data.

Here, V_{sim} is the velocity obtained from COMSOL and V_{ANN} is the data predicted data, and n denotes the total tested datapoints [31]. Elevated precision is associated with reduced ~~Mean Absolute Percentage Error~~ (MAPE), ~~Root Mean Square Error~~ (RMSE), and ~~Mean Bias Error~~ (MBE) values. The Artificial Neural Network (ANN) model underwent evaluation using MAPE as a classification metric, with a MAPE of less than 10% indicating a high degree of accuracy. After this classification, the mean MAPE was calculated for each model, enabling the determination of a model suitable for turbines of differing dimensions.

3.5. Data Processing

In this research investigation, a thorough correlation analysis was conducted utilizing the Python programming language to explore the associations between two pivotal variables: the turbine's inlet velocity and velocities at various designated points and sections within the tur-

bine system, as illustrated in Figure 4. The primary aim of this study is to ascertain the underlying nature of the relationship between the variables. To achieve this objective, multiple datasets obtained from simulation-based studies and Artificial Neural Networks (ANN) were subjected to advanced statistical analysis to compute the correlation coefficient. Specifically, Pearson's correlation coefficients were computed in this context to assess both the strength and direction of the relationships that exist among these variables.

4. RESULTS

4.1. Simulation

From Figure 6 we know that the velocity just before the turbine (V1) is equal to the velocity after the turbine (V2). This voids the postulates stated by the Actuator Disk theory as no reduction in the velocity is seen after the turbine. This may be attributed to the behavior of air through the turbine. It can also be seen that throughout the turbine there is a gradient of velocity. This would suggest that the thrust throughout the turbine would be different. The curvature of the velocity suggests that multiple types of velocities are present in the turbine region and it imperative that all these types of velocities be considered.

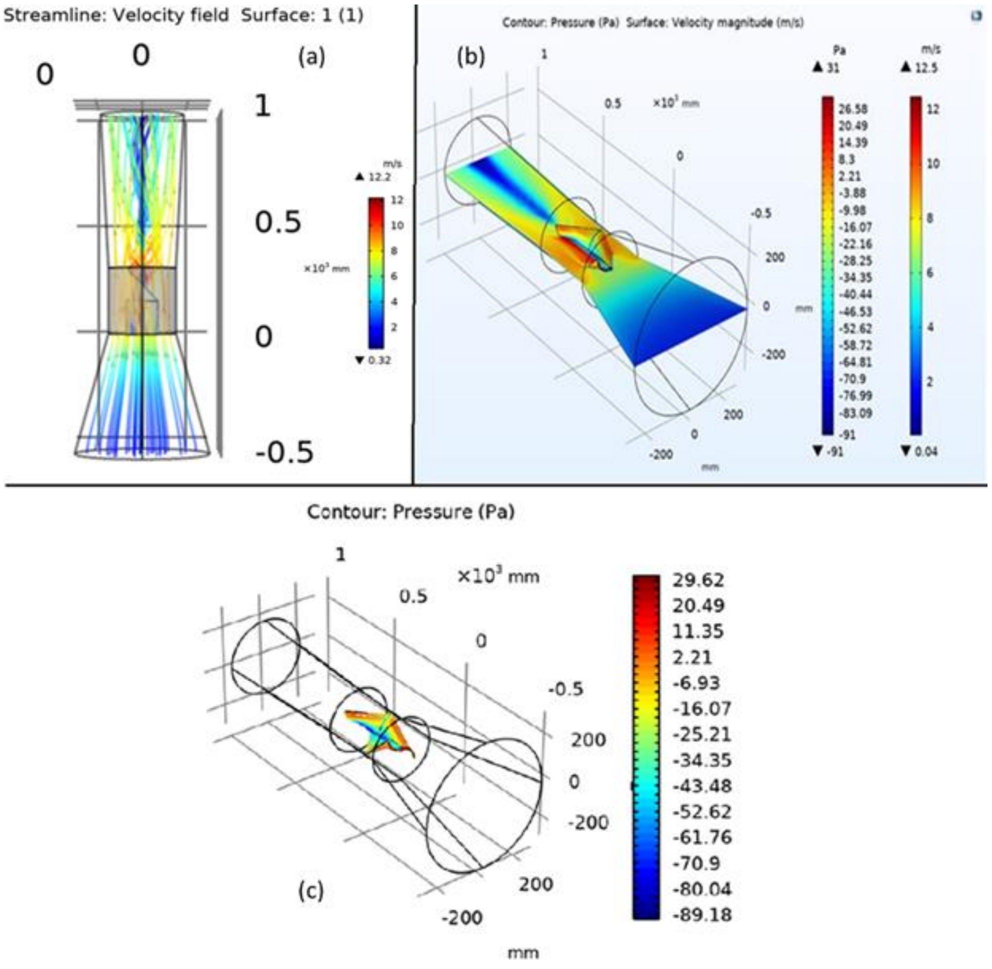

FIGURE 6: (a) Streamline velocity depiction through the ducted Horizontal Axis-Helical Wind Turbine system. (b) Velocity and Pressure relation on a cut plane through the ducted Horizontal Axis-Helical Wind Turbine system. (c) Pressure on the turbine defining the velocity profile across the turbine.

The velocities to be considered include the axial velocity (Va) and tangential velocity (Vc). The Va and Vc lie within the turbine, where Va is seen near the central axis and Vc between the central axis and the underside of the blade edge. To understand the profile better it was necessary that more data be obtained about the velocity profile. The confirmation of the same can be seen in the next section.

4.2. Artificial Neural Network

From figure 7 we know that a velocity gradient is present across the diameter and the length of the turbine. It can also be noted that the velocity profile at the outermost edge of the turbine is seen dropping. However, the case for the velocity profile near the axis of the turbine is seen decreasing until the first 20% of the turbine followed by a sharp increase. Later the velocity profile is seen stabilizing. It can also be seen that the velocity profile in the mid-section of the turbine shows a very marginal increase in the velocity profile followed by a very small decrease in the velocity to the end of the turbine. The behavior of the wind near the axis is such due to the stagnation point being very close to the axis in the initial section of the turbine and the turbine acting as a funnel beyond the 10% of the turbine.

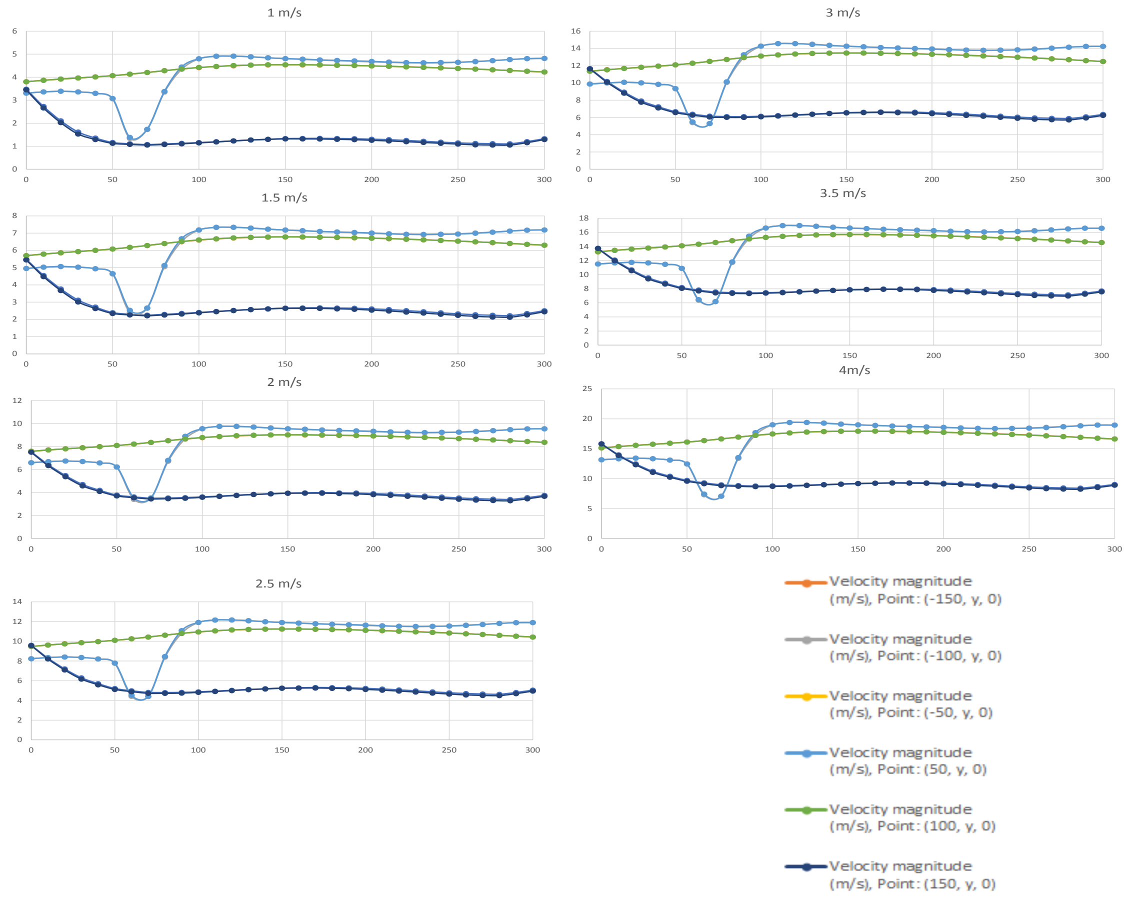

FIGURE 7: Velocity gradient throughout the turbine at various points and velocity.

Using the inputs from the simulation as targets the system was trained nine times to verify the accuracy of data stream. The use of data from the simulation makes it easy to acquire data which was previously impossible to attain and provides information with regards to the interaction of wind with the turbine and its aerodynamics. This also proves that similar irregularly shaped turbines can be analyzed for their aerodynamics at a range of velocities based on basic inputs from simulations. It has been seen that the tick and trial method has been effective and has helped in the appropriate neuron. In this the velocity range has been tested and predicted in case where the incoming wind velocity was 4 m/s and 6 m/s respectively. Table 1 talks about the error in the data generated from ANN.

TABLE 1: Simulation data evaluation metrics.

Points	MAPE	MBE	RSME
150	2.41%	0.18 m/s	0.32 m/s
100	1.53%	0.01 m/s	0.30 m/s
50	4.02%	0.86 m/s	0.45 m/s
-50	2.78%	0.51 m/s	0.17 m/s
-100	1.77%	0.37 m/s	0.21 m/s
-150	2.36%	0.26 m/s	0.05 m/s

Further, to understand the difference between the simulation based and the ANN based data the velocity profile at each of the diameter section was plotted. It can be seen from figure 8 that both the simulated and predicted data are well in agreement. This is well in agreement with the MAPE obtained earlier.

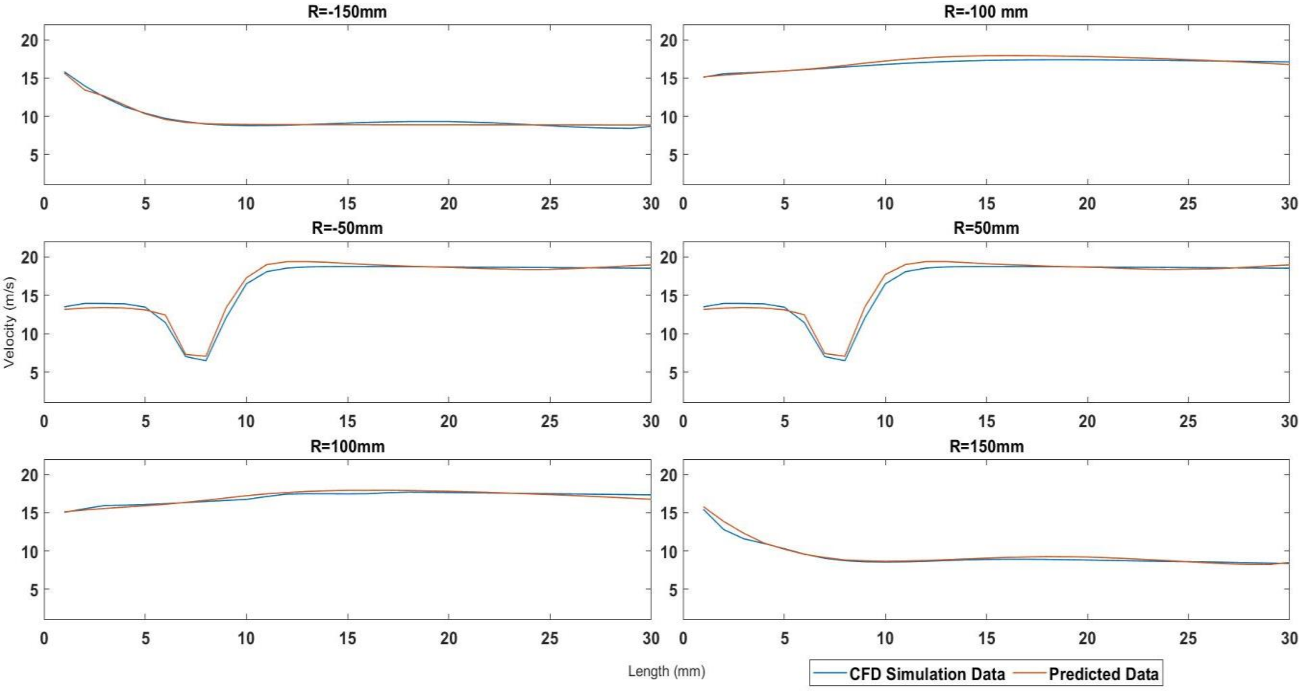

Figure 8: Simulation based and predicted data co-relation.

4.3. Pearson’s Correlation

The primary aim of the correlation analysis was to assess and quantify the relationships between the turbine's inlet velocity and the magnitudes of velocity at distinct positions along the diameter of the turbine blade. This analysis utilized a heatmap diagram to visually represent these connections as shown in figure 9.

Interestingly, the analysis revealed a strong and statistically significant correlation between the inlet velocity and the velocity magnitudes at various positions across the turbine blade's diameter. This finding suggests that changes in the inlet velocity have a direct and measurable impact on the velocity magnitudes at specific locations of the turbine blade. The study also suggests that the velocity is inversely related with the incoming velocity and directly related at certain locations in the turbine. Consequently, modifications in the inlet velocity, whether an increase or decrease, correspondingly lead to alterations in the velocity magnitudes at these positions.

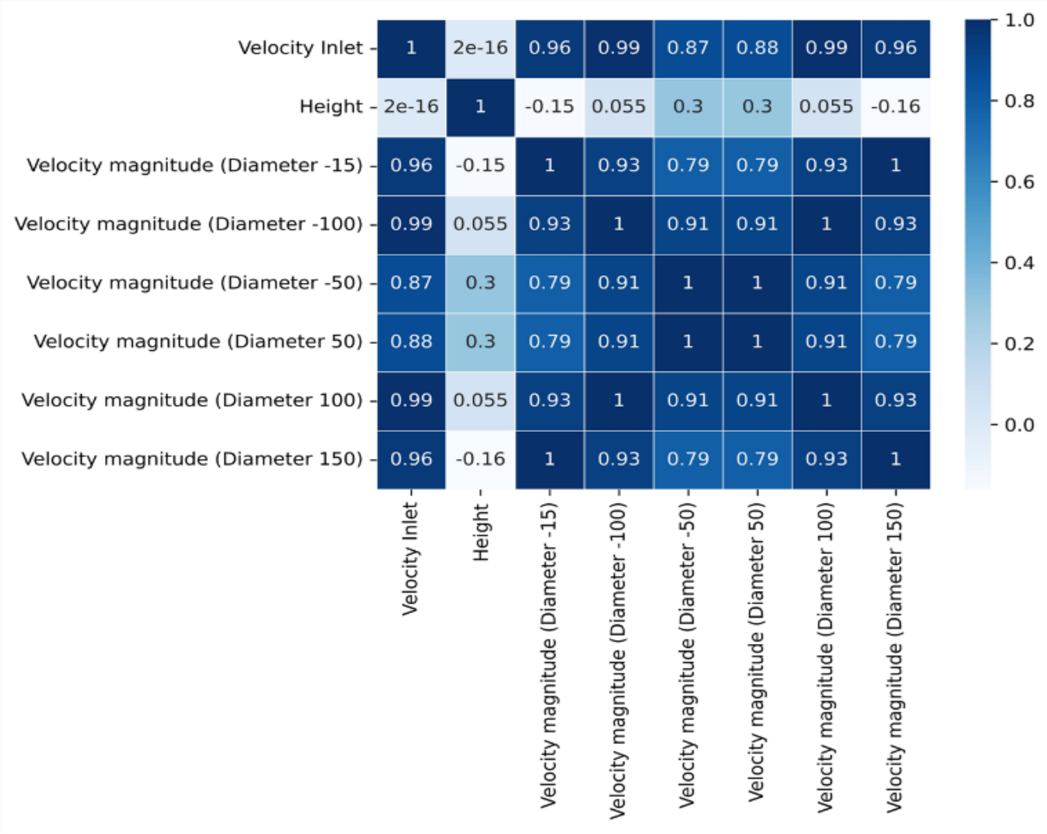

FIGURE 9: Statistical evaluation of the relationship between the inlet velocity and velocity at different segments of the turbine.

Moreover, the velocity magnitudes at discrete points spanning the diameter of the turbine blade exhibit noticeable interrelationships. This observation indicates that changes in velocity magnitude at one point tend to be mirrored by similar changes at other points along the blade's diameter. A consistent pattern of variation in velocity magnitude prevails across the entirety of the blade's diameter.

4.4. New Mathematical Model

Figure 10 introduces the indexes used at various locations. Here, the ambient pressure is noted as P0, V1 is the velocity just before the turbine and P1 is the pressure just before the turbine, P2 and V2 are the downwash pressure and velocity of the turbine, respectively. Finally, the P3 and V3 are the exit pressure and velocity, respectively. To understand more about the flow, we need to know the type of the flow, which in this condition is the incompressible flow. As the flow is incompressible, we know that the continuity equation can be applied as there is no change in the density. The velocity range can be known from Figure 3, which shows the velocity at different points.

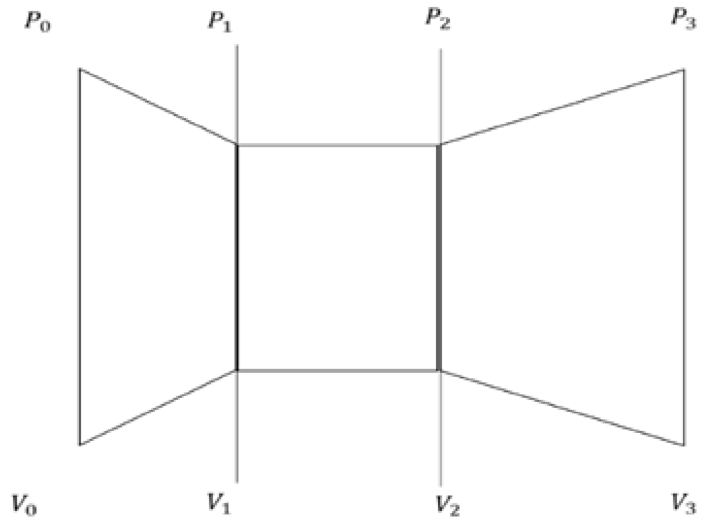

Figure 10: Pressure and velocity relation in an empty diffuser.

Therefore,

$$\boldsymbol{P + \frac{1}{2}\rho V^2 = Constant} \text{......................................} \quad \text{(xi)}$$

$$\boldsymbol{P_1 + \frac{1}{2}\rho V_1^2 = P_2 + \frac{1}{2}\rho V_2^2 = P_3 + \frac{1}{2}\rho V_3^2}$$

Figure 11 shows the direction of the wind flow with respect to the turbine and the velocity triangle. As we have established earlier, we know that we must consider the velocity triangle in this situation. And hence the equation becomes:

Figure 11 shows the direction of the wind flow with respect to the turbine and the velocity triangle. As we have established earlier, we know that we must consider the velocity triangle in this situation. And hence the equation becomes:

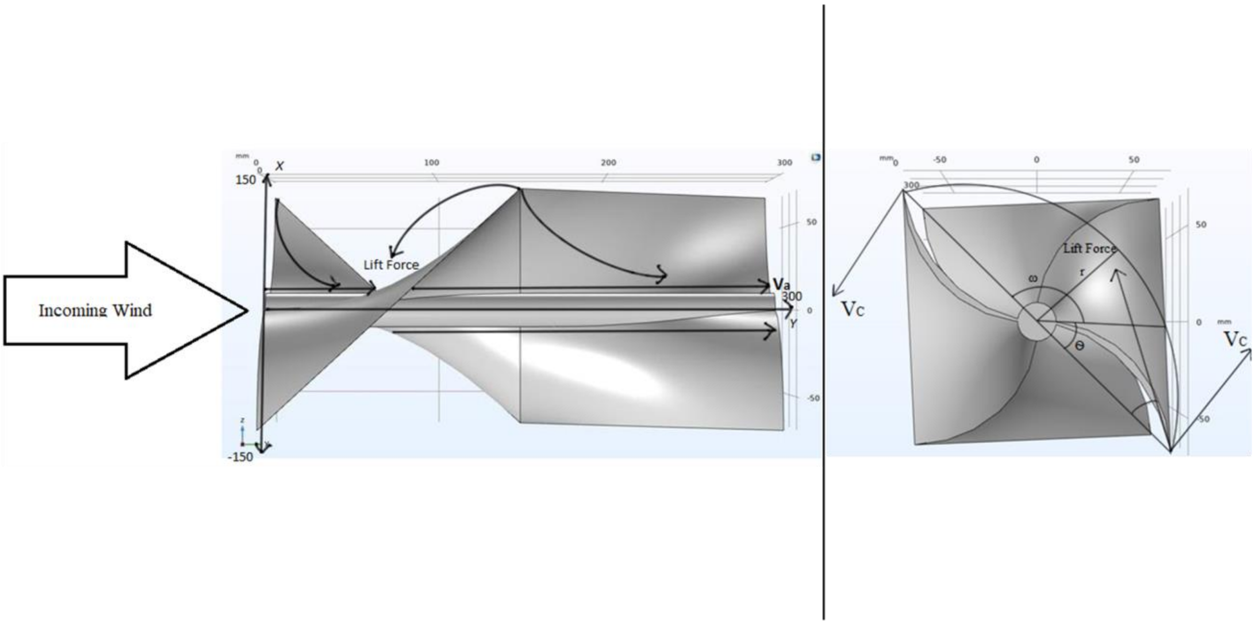

Figure 11: Blade velocity and force diagram. Here, ω is the rotation, r is the radius, θ is the angle.

$$\boldsymbol{P + \frac{1}{2}\rho(v_{resultant})^2 = Constant}$$

$$Where\ v_{resultant} = \sqrt{v_c + v_a}$$

v_c= tangential velocity
v_a=axial velocity
But,

$$\boldsymbol{v_c = r\omega}$$

Substituting the value of $v_c$in equation 1, where r = radius (meter) and ω = angular velocity, (rad/sec) which corresponds to the radius of the HHAWT, we get,

$\boldsymbol{P + \frac{1}{2}\rho\left(\sqrt{r\omega + v_a}\right)^2 = Constant}$……….. (xii)

However, looking at the simulations we understand that we have different velocities at different radial points of the turbine. In which case the average of $v_c$would be considered. Hence,

Average $v_{c=} \frac{v_{c1}+v_{c2}+v_{c3}\ldots\ldots\ldots v_{cn}}{n}$

Therefore,

$$\boldsymbol{Power} = \frac{1}{2}\boldsymbol{A\rho}\left(\frac{\sum_i^{30}\left[\sqrt{\left\{\frac{\sum_i^4 v_c}{4}\right\}+\left\{\frac{\sum_i^2 v_a}{2}\right\}}\right]}{30}\right)^3 \ldots\ldots\ldots\ldots\ldots\ldots \text{(xiii)}$$

To calculate the pressure profile of the turbine the following equation can be used.

$$\boldsymbol{P} + \frac{1}{2}\boldsymbol{\rho}\left(\frac{\sum_i^{30}\left[\sqrt{\left\{\frac{\sum_i^4 v_c}{4}\right\}+\left\{\frac{\sum_i^2 v_a}{2}\right\}}\right]}{30}\right)^2 = \boldsymbol{Constant}$$

For the calculation of the torque the equation suggested is:

$$\boldsymbol{Torque} = \frac{1}{2}\boldsymbol{A\rho C_T}\left(\frac{\sum_i^{30}\left[\sqrt{\left\{\frac{\sum_i^4 v_c}{4}\right\}+\left\{\frac{\sum_i^2 v_a}{2}\right\}}\right]}{30}\right)^3 \ldots\ldots\ldots\ldots\ldots\ldots \text{(xiv)}$$

To calculate the lift-force the formula used is:

$$\mathrm{F} = \mathrm{C_L A q}\left(\frac{\sum_i^{30}\left[\sqrt{\left\{\frac{\sum_i^4 v_c}{4}\right\}+\left\{\frac{\sum_i^2 v_a}{2}\right\}}\right]}{30}\right)^2 \ldots\ldots\ldots\ldots\ldots \text{(xv)}$$

Here, C_L is the coefficient of lift, A is the area and q are the dynamic pressure.

Based on the equation we have and the behavior of the turbine we can say that the energy balance equation changes to the following:

$$\Delta E = \frac{P_1}{\rho} + \frac{V_1^2}{2} - \left[\left(\frac{P_2}{\rho} + \frac{(V_1)^2}{2}\right) + \frac{1}{2}\rho A V_1^3\right] \ldots\ldots\ldots\ldots\ldots \text{(xvi)}$$

From the energy balance equation, we know that the velocity of the wind after the turbine will be the same. This suggests that the Kinetic energy of the velocity is conserved. The development of this energy balance equation suggests that no back force or negative pressure on the system would be seen. This makes the energy recovery system applicable for various exhaust systems.

5. CONCLUSION

The conclusion necessitates the formulation of a novel mathematical model to account for the unique characteristics of the High Hub Axial Wind Turbine (HHAWT). The traditional Blade Element Momentum (BEM) theory, along with the Actuator Disk Theory, is no longer applicable for assessing the turbine's performance due to its novel features. Based on the Computational Fluid Dynamics (CFD) data, it is evident that the turbine exhibits a velocity gradient across its structure, encompassing distinct types of ve-

locities, such as tangential and linear velocity. Notably, the turbine functions akin to a funnel, amplifying the velocity at its central axis by a factor of 3. This velocity differentiation throughout the turbine significantly impacts lift at various locations within the turbine.

The proposed new mathematical model can pave the way for exploring unconventional turbine shapes and designs, enabling the development of turbines with enhanced mechanical power extraction capabilities. Furthermore, this research can lead to the formulation of innovative CFD procedures for analyzing turbine performance computationally. However, before integrating the model with CFD simulations, it is imperative to refine the equations and particularly define the coefficient of lift to suit such turbines accurately. This step is crucial to ensure the accuracy and precision of the forthcoming analyses and simulations.

REFERENCE

1. Bridge G, Özkaynak B, Turhan E. Energy infrastructure and the fate of the nation: Introduction to special issue. Energy Res Soc Sci. 2018;41: 1–11. doi:10.1016/j.erss.2018.04.029
2. Al-Kayiem HH, Aja OC. Historic and recent progress in solar chimney power plant enhancing technologies. Renew Sustain Energy Rev. 2016;58: 1269–1292. doi:10.1016/j.rser.2015.12.331
3. Sabzevari A. Performance Characteristics of Concentrator-Augmented Savonius Wind Rotors. Wind Eng. 1977;1: 198–206. Available: http://www.jstor.org/stable/43749084
4. Ohya Y, Karasudani T. A shrouded wind turbine generating high output power with wind-lens technology. Energies. 2010;3: 634–649. doi:10.3390/en3040634
5. Chong WT, Fazlizan A, Poh SC, Pan KC, Hew WP, Hsiao FB. The design, simulation and testing of an urban vertical axis wind turbine with the omni-direction-guide-vane. Appl Energy. 2013;112: 601–609. doi:10.1016/j.apenergy.2012.12.064
6. Allaei D, Andreopoulos Y. INVELOX: Description of a new concept in wind power and its performance evaluation. Energy. 2014;69: 336–344. doi:10.1016/j.energy.2014.03.021
7. Han W, Yan P, Han W, He Y. Design of wind turbines with shroud and lobed ejectors for efficient utilization of low-grade wind energy. Energy. 2015;89: 687–701. doi:10.1016/j.energy.2015.06.024
8. Chong WT, Muzammil WK, Fazlizan A, Hassan MR, Taheri H, Gwani M, et al. Urban Eco-Greenergy™ hybrid wind-solar photovoltaic energy system and its applications. Int J Precis Eng Manuf. 2015;16: 1263–1268. doi:10.1007/s12541-015-0165-3
9. Aja Ogboo Chikere HHA-K and ZAAK. Review on the Enhancement Techniques and Introduction of an Alternate Enhancement Technique of Solar Chimney Power Plant. J Appl Sci. 2011;11: 1877–1884.
10. Chilugodu N, Yoon YJ, Chua KS, Datta D, Baek JD, Park T, et al. Simulation of train induced forced wind draft for generating electrical power from Vertical Axis Wind Turbine (VAWT). Int J Precis Eng Manuf. 2012;13: 1177–1181. doi:10.1007/s12541-012-0156-6
11. Mann HS, Singh PK. Kinetic Energy Recovery from the Chimney Flue Gases Using Ducted Turbine System. Chinese J Mech Eng (English Ed. 2017;30: 472–482. doi:10.1007/s10033-017-0090-8
12. Mann HS, Singh PK. Effect of number of blades in ducted turbine system on kinetic energy extraction from chimney flue gases – benchmarking with wind energy system. J Mech Sci Technol. 2018;32: 5443–5455. doi:10.1007/s12206-018-1042-7
13. Thangavelu SK, Goh CY, Sia C V. Design and Flow Simulation of Concentrator Augmented Wind Turbine. IOP Conf Ser Mater Sci Eng. 2019;501. doi:10.1088/1757-899X/501/1/012041
14. Puttichaem W, Putivisutisak S, Boonyongmaneerat Y, Vadhanasindhud P. Early development of a shaftless horizontal axis wind turbine for generating electricity from air discharged from ventilation systems. Int J Energy Res. 2020;2019: 1–11.

doi:10.1002/er.6221
15. Ibrahim M, Alsultan A, Shen S, Amano RS. Advances in Horizontal Axis Wind Turbine Blade Designs: Introduction of Slots and Tubercle. J Energy Resour Technol. 2015;137. doi:10.1115/1.4030399
16. Beyhaghi S, Amano RS. Improvement of Aerodynamic Performance of Cambered Airfoils Using Leading-Edge Slots. J Energy Resour Technol Trans ASME. 2017;139: 1–8. doi:10.1115/1.4036047
17. Franco JA, Jauregui JC, Toledano-Ayala M. Optimizing wind turbine efficiency by deformable structures in smart blades. J Energy Resour Technol Trans ASME. 2015;137: 1–8. doi:10.1115/1.4030445
18. Van Bussel GJW. The science of making more torque from wind: Diffuser experiments and theory revisited. J Phys Conf Ser. 2007;75. doi:10.1088/1742-6596/75/1/012010
19. Rogowski K, Hansen MOL, Lichota P. 2-D CFD Computations of the Two-Bladed Darrieus-Type Wind Turbine. J Appl Fluid Mech. 2018;11: 835–845. doi:10.18869/acadpub.jafm.73.247.28383
20. Troldborg N, Sørensen JN, Mikkelsen R. Actuator line simulation of wake of wind turbine operating in turbulent inflow. J Phys Conf Ser. 2007;75. doi:10.1088/1742-6596/75/1/012063
21. Sørensen JN. The general momentum theory. Research Topics in Wind Energy. 2016. doi:10.1007/978-3-319-22114-4_4
22. Mathew S. Wind Energy: Fundamentals, Resource Analysis and Economics. 2006.
23. Frandsen S, Barthelmie R, Pryor S, Rathmann O, Larsen S, Højstrup J, et al. Analytical modelling of wind speed deficit in large offshore wind farms. Wind Energy. 2006;9: 39–53. doi:10.1002/we.189
24. Larsen GC. A Simple Wake Calculation Procedure. Risø-M. 1988;No. 2760: 58. Available: http://orbit.dtu.dk/ws/files/55567186/ris_m_2760.pdf
25. Elliott DL. Status of wake and array loss research. Proc 21st Am Wind Energy Assoc Conf Wind. 1991; 224–227.
26. Sedaghatizadeh N, Arjomandi M, Kelso R, Cazzolato B, Ghayesh MH. Modelling of wind turbine wake using large eddy simulation. Renew Energy. 2018;115: 1166–1176. doi:10.1016/j.renene.2017.09.017
27. Suheel SZ, Fazlizan A. Workability of a new kinetic energy recovery system proven mathematically. AIP Conf Proc. 2021;2339. doi:10.1063/5.0044199
28. Acarer S, Uyulan Ç, Karadeniz ZH. Optimization of radial inflow wind turbines for urban wind energy harvesting. Energy. 2020;202. doi:10.1016/j.energy.2020.117772
29. Wang Q, Wang J, Hou Y, Yuan R, Luo K, Fan J. Micrositing of roof mounting wind turbine in urban environment: CFD simulations and lidar measurements. Renew Energy. 2018;115: 1118–1133. doi:10.1016/j.renene.2017.09.045
30. Šarkić Glumac A, Hemida H, Höffer R. Wind energy potential above a high-rise building influenced by neighboring buildings: An experimental investigation. J Wind Eng Ind Aerodyn. 2018;175: 32–42. doi:10.1016/j.jweia.2018.01.022
31. Mubiru J. Predicting total solar irradiation values using artificial neural networks. Renew Energy. 2008;33: 2329–2332. doi:10.1016/j.renene.2008.01.009
