# Supplementary material for: Mathematical modeling and performance evaluation of Ducted Horizontal-axis Helical Wind Turbines: Insights into aerodynamics and efficiency
Source: PLoS One. 2024 Jun 17;19(6):e0303526. doi: 10.1371/journal.pone.0303526 (PMC11182528; doi:10.1371/journal.pone.0303526)
Supplement: Attachment — Submitted filename: Response to Reviewer.docx [file pone.0303526.s003.docx]

The authors of the paper are thankful to the reviewers. The comments, suggestions and reviews have been understood and implemented in the paper. We hope these suggestions would aid in further improve the quality of the paper and help make an iota of difference in the field.

Reviewer 1

| Review | Response |
| --- | --- |
| Could you provide insights into the computational resources required for these simulations? How scalable is the model to handle larger or more complex turbine designs? | 1. The device used for the initial simulation was Dell Latitude 13 7320, 11^th^ Generation i7-Processor, 16GB DDR4 RAM. Later for further curation of data and results the system used was Intel Xenon CPU-E5-26900, 32GB DDR3 RAM, with 8 cores, Logical Processor 16. 2. As of now due to the limitation of computational power of the systems available scalability of the system was not tested. 3. However, in the future the authors want to test the scalability and the application of the same for the same. 4. When it comes to complex turbine designs, the authors have evidence to believe that as long as the aerodynamic interactions and behaviour of the complex turbine is same the simulation as well as the mathematical model shall be applicable. |
| I recommend considering a more descriptive heading for “2. Theory Review” perhaps something like “Theoretical Overview of Wind Turbine Performance” to better reflect the content and purpose of this section. | We thank you for the suggestion and accept and implemented the suggestion. |
| Consider relocating Figure 2 to the “3. Methodology” section to improve the flow of information. | We thank you for the suggestion and accept and implemented the suggestion. |
| The methodology relies on a control volume assumption. How sensitive is the model to variations in the shape and size of the control volume? It wasn’t clear to me if different control volume configurations have been explored, and if so, what impact do they have on turbine performance analysis. | The authors have tested the system at different control volumes and the trends of the flow were noted and described in figure 7. However, due to constraints in the computational power the authors could not evaluate the performance based on shape of the control volume. For the same the authors have some experimental data. |
| In section 3.3, the fluid pressure is denoted as “p”; in the text, but it doesn’t appear in any equations. Consider using the “rho” Greek letter to denote fluid density for clarity and consistency in notation. | We thank you for the suggestion and accept and implemented the suggestion. |
| How sensitive is the model to variations in temperature, and was the impact of temperature changes on fluid properties considered in the analysis? | The temperature parameter has not been considered by the authors as temperature would not affect the properties of the fluid unless the temperature fluctuation would be in a range of ± 10^○^C. The impact of temp is negligible. |
| How does the simulation model account for dynamic changes in wind conditions? Are there considerations for transient simulations to capture the turbine’s response to varying wind speeds? | The current system designed has a unidirectional flow from the inlet at the bottom to the outlet at the top, owing to this the dynamic changes in the wind direction were not taken into consideration. However, an effort to capture the response of the turbine turbulence created by a fan at the inlet was made but the computational requirements were very high. |
| The duct material is specified as Polyvinyl Chloride (PVC). Could you discuss how the choice of duct material influences the simulation results? Were material properties, such as roughness, taken into account in the analysis? | 1. After the computational study a prototype was developed in order to study the experimental performance of the system. The material used for the developed prototype was PVC, hence, making it the choice. 2. Yes, the material property of roughness was taken into account during the simulation. |
| Some figures in the text appear to be written in lowercase. | We thank you for the suggestion and accept and implemented the suggestion. |
| Some sections of the text use the first person (“we”). Consider revising these instances to the third person for a more formal and objective tone in scientific writing. | We thank you for the suggestion and accept and implemented the suggestion. |

Reviewer 2

The authors of the paper are thankful to the reviewers. The comments, suggestions and reviews have been understood and implemented in the paper. We hope these suggestions would aid in further improve the quality of the paper and help make an iota of difference in the field.

| Review | Response |
| --- | --- |
| The motivation of the use of this kind of machine is not well described. What is the advantage in comparison with a classical ducted wind turbine? What is the advantage in comparison with a helical wind turbine with a vertical axis? | The motivation for the development of this technology has been added to the introduction section in the second paragraph. The advantage of such device over classical wind turbines has also been explained here. |
| The performance of the machine are not shown, the CP-lambda curves are not evaluated. | The C_p_-Lambda curve is an important graph in understanding the performance of the wind turbine. However, due to the following reasons the C_p_-Lambda curve was not included:   1. The study conducted was aimed at understanding the aerodynamics of the turbine and the behaviour of wind around the turbine. 2. The current computing system available lacks the computational power to simulate such heavy simulations. |
| The simple mechanical balance on ducted wind turbines are impenetrable, and a significant original contribution lacks. | 1. As the system has one point of inlet and one point of outlet and is enclosed within a duct, there is no mixing or impact of the free-flowing wind. Hence, the energy balance does not reflect the impact of the same considering that the free-flowing wind is impenetrable into the system. 2. The novelty of the research include: a. The configuration of the system designed. b. The newly derived equation. c. The novel working theory |
| The bibliography review on ducted wind turbines misses important and recent works revealing the real potential of this kind of devices. | Recent publication has been added as per the suggestion. |
| The use of an ANN is not motivated. | The motivation behind using ANN was that it was a computationally affordable method to generate large amount of data to understand the velocity profile trends. |
